# Gastric Carcinoma in Young Patients and Its Clinicopathological Characteristics and Prognosis

**DOI:** 10.1155/2020/7378215

**Published:** 2020-07-13

**Authors:** Bhushan Sandeep, Xin Huang, Yuan Li, Long Mao, Ke Gao, Zongwei Xiao

**Affiliations:** ^1^Department of Surgery, Chengdu Second People's Hospital, Chengdu, Sichuan 610017, China; ^2^Department of Anesthesiology, Chengdu Second People's Hospital, Chengdu Sichuan 610017, China

## Abstract

**Background:**

Gastric cancer is usually an age-related disease and mostly diagnosed after the sixth decade of life, though it may also be diagnosed earlier.

**Objective:**

The aim of this study is to explore the clinicopathological characteristics and prognosis of gastric carcinoma in young patients.

**Methods:**

A total of 1379 patients younger than 75 years histologically diagnosed with primary gastric carcinoma underwent gastrectomy. Patients were categorized into three groups based on their age which included young age group (≤40 years), middle-aged group (age 41-60 years), and elderly group (age 61-75 years). The young age group was further subdivided into two groups: Group A (age ≤35 years) and Group B (age 35-40 years). The analysis of the clinicopathological characteristics and prognosis followed thereafter.

**Results:**

Females predominate in young group (*p* < 0.001). A significantly higher undifferentiated histological pattern was found in the young age group from the other two groups (*p* < 0.001). Tumor location in the lower third of the stomach was significantly higher in the young group than the other groups (*p* < 0.001). T4 stage was common in young patients similar to the middle and old age group (*p* = 0.049). Distal gastrectomy was performed more in the young age group rather than the middle and old age groups with the following percentage ratios: young group 74.5% (123/165), middle age group 59.9% (429/716), and old age group 52.2% (260/498) (*p* < 0.001). The 5-year overall survival rate of the young, middle, and old age groups were 46%, 48%, and 39%, respectively, whereas the 5-year overall survival rates of the subgroups of young patients, Group A and Group B, were 33% and 49%, respectively. Multivariate analysis revealed that independent negative prognostic factors were as follows: tumor location (L), *p* = 0.016, OR = 0.795, 95%CI = [0.659; 0.959]; larger tumor size, *p* = 0.026, OR = 1.727, 95%CI = [1.067; 2.797]; resection margin, *p* < 0.001, OR = 2.167, 95%CI = [1.751; 2.682]; tumor stage (T4), *p* < 0.001, OR = 2.572, 95%CI = [1.709; 3.870]; and nodal involvement N1, *p* = 0.005, OR = 1.506, 95%CI = [1.123; 2.020]; N2, *p* < 0.001, OR = 1.708, 95%CI = [1.289; 2.263]; and N3, *p* < 0.001, OR = 2.986, 95%CI = [2.314; 3.854].

**Conclusion:**

The young age groups of patients were predominantly female and had a higher proportion of poorly differentiated and undifferentiated type of tumor; moreover, patients aged <35 years had a poor prognosis. In addition, gastric cancer can occur in patients less than 30 years old, and symptoms suggestive of gastric cancer should be investigated aggressively; therefore, a close scrutiny and monitoring should be done in younger patients especially those associated with high-risk factors which could indicate the presence of the disease at an early stage.

## 1. Introduction

Gastric cancer is the second most frequent cause of cancer-related deaths, with the highest incidence in the middle-aged and elderly populations [1, 2]. The rate of gastric carcinoma has increased in young patients over the past few decades [3, 4]. Though gastric carcinoma is rare in young people, it commonly shows a more aggressive biological behavior with a worse prognostic result [5, 6]. Nearly 5-15% of the patients with gastric carcinoma are aged <40 years, and only 1-2% of the patients are aged <30 years [5, 7, 8]. In young patients, the clinical outcome still remains a controversy despite some studies have indicated a poor prognosis in young patients [9]. Other reports showed a similar survival rate in both young and elderly patients [10–12]. However, some studies have concluded a better prognosis for young patients [13]. Most of studies included only elderly patients creating an inconclusive result to all the age groups. Researches about young patients were very less or included a small number of patients with a result outcome having contradiction. Most of the patients with gastric carcinoma were diagnosed at an advanced stage thereby affecting the overall survival rate of the patients. In this study, we aim to explore the clinicopathologic characteristics and elucidate the prognostic factors of gastric cancer patients below the age of 40 years or younger who underwent gastrectomy.

## 2. Materials and Methods

### 2.1. Inclusion and Exclusion Criteria

The data of consecutive patients, who underwent gastrectomy from January 2007 to December 2017 in the Department of General Surgery, were retrospectively collected and analyzed. The protocol was approved by the Chengdu Second People's Hospital Clinical Research Ethics Committee, and the parents of all subjects provided written informed consent during admission. The inclusion criteria were (1) histologically proven gastric cancers, (2) distal, total, or proximal gastrectomy, and (3) a complete available medical record. The exclusion criteria were remnant stomach cancer, nonepithelial malignant tumor, gastrointestinal stromal tumor, lymphoma, carcinoid tumor, small cell carcinoma, squamous cell carcinoma, adenosquamous cell carcinoma, secondary carcinoma, and an age above 75 years due to lack of follow-up information were excluded from the study. We divided these 1379 patients who fulfill the criteria into 3 groups according to the ages: the young age group (YG) (≤40 years); the middle age group (MG) (age 41-60 years); and the elderly group (OG) (60-75 years). The young group was further subdivided into two groups: Group A, (age ≤35 years) and Group B (age 35-40 years).

### 2.2. Clinicopathologic Characteristics

The clinicopathological characteristics were reviewed from the Gastric Cancer Database of our Hospital, with regards to each patient's age, sex, tumor size, tumor location, Bormann macroscopic type, differentiate degree, T stage, N stage, TNM stage, resection margin (R0, R1, and R2), resection patterns (distal gastrectomy, total gastrectomy, or proximal gastrectomy), and postoperative hospital stay (days). Laparoscopic-assisted distal gastrectomy was applied for patients whose clinical staging carries a lower chance of lymph node metastasis. Traditional open gastrectomy was performed in patients whose clinical staging carries a higher chance of lymph node metastasis with a significantly higher number of lymph nodes removed compared to the laparoscopic approach. Resection margin (R0, R1, and R2) and the Bormann macroscopic type (Types 0-4) were evaluated according to the Japanese gastric cancer treatment guideline [14]. R0 is defined as curative resection while R1 with microscopic tumor residual and R2 with macroscopic tumor residual. Both the differentiated degree and tumor stage were evaluated according to the seventh edition of the American Joint Committee on Cancer Staging System [15].Signet-Ring cell carcinoma of stomach is a malignant tumor that is usually seen in adults over 30 years of age. Our study reports indicate that 30% of young population below 40 years belong to this type, and usually women of younger age are affected by SRCC of stomach which is more common (58%). The longstanding untreated stomach infection by bacterium *H. pylori* was the most important predisposing factor of SRCC.

### 2.3. Treatment

The surgical treatment strategy was according to the Japanese classification of gastric carcinoma (JCGC) and performed with curative intent [16]. Depending on the location and size of the tumor, the surgeon performed distal, total, or proximal gastrectomy. Lymphadenectomy included D1, D1+, D2, or D2+ nodal resection. Billroth-1 and Billroth-2 anastomoses were commonly used for distal gastrectomy while the Roux-en-Y anastomosis was selectively performed, esophagojejunal Roux-en-Y anastomosis was used for total gastrectomy, and esophagogastrostomy was used for the proximal gastrectomy.

### 2.4. Follow-Up Information

Patients were regularly followed up by outpatient visit, mail, or telephone, and the information was updated until 20 December 2018. The follow-up rate, median follow-up duration (months), and overall survival outcomes were analyzed. The reasons for the patient's lack of follow-up were predominantly refusal of the outpatient visit or a change in the telephone number and address.

### 2.5. Statistical Analysis

Statistical analysis was performed with SPSS 19.0. Continuous data with normal distribution were adopted the one-way ANOVA test, while the Mann–Whitney *U* test was used for the data not conforming to normal distribution. Continuous data are presented as mean ± standard deviation. For categorical data, the Chi-square test or Mann–Whitney *U* test was used. The Kaplan–Meier curves (log-rank test) were used for analyses of survival outcome, and the log-rank test was carried out to test the statistical significance. Multivariate analysis adopted the Cox regression with the forward stepwise method. A two-sided *P* value of less than 0.05 was considered statistically significant.

## 3. Results

### 3.1. Patient Demographics

Clinicopathological details and surgical results of gastric carcinoma in young, middle, and old patients are shown in Tables 1 and 2. The age, median age, sex, male-to-female ratio, number with percentage of patients, occurrence of tumor, and tumor resection pattern in all three groups are shown in Tables 1 and 2. Distal gastrectomy was performed more in YG than in MG or OG with the following percentage ratios: YG 74.5% (123/165), MG 59.9% (429/716), and OG 52.2% (260/498) (*P* < 0.001). The tumor stage in all three groups is shown in Tables 1 and 2. As in the MG and the OG, T4 stage disease at presentation was more common than the lesser stages (*p* = 0.049). A significantly higher proportion of undifferentiated histological pattern was found in the YG with respect to the other two groups. The % ratio was as follows: YG, 150 patients (91%); MG, 616 patients (86.0%), and OG, 398 patients (80%) (*P* < 0.001). However, the mean number of harvested lymph nodes in the YG was similar in comparison with the other two groups (YG: 23.6 ± 12.7, MG: 24.3 ± 13.9, and OG: 22.2 ± 13.6), which was statistically significant (*P* = 0.028). The mean number of postoperative stay is comparatively less in YG as compared to MG or OG (data showed, YG 9.8 ± 2.5, MG 10.5 ± 4.5, and OG 11.5 ± 5.6) (*p* < 0.001). There were no significant differences found in YG in comparison with the other two groups in terms of resection degree, surgical duration, Bormann type, number of positive lymph nodes, and TNM staging. Clinicopathological details and surgical results of gastric carcinoma in YG are shown in Tables 3 and 4. The YG was further divided into two subgroups, A and B. Group A comprised of 46 patients (age ≤35 years) in which 21 were male and 35 were female, and Group B comprised of 119 patients (age 35-40 years) in which 61 were male and 58 were female. The mean age of Group A and Group B patients was 27 ± 3.8 and 36.4 ± 2.7 years, which is statistically significant (*P* < 0.001). The male-to-female ratios in Group A and Group B were 1 : 1.6 and 1 : 1, respectively, with a higher number of females in Group A compared with Group B. With respect to the occurrence and location of gastric tumors, it was frequently seen at the lower part (third) of the stomach in both the age groups (89% vs. 77.3%). The tumors arising from the upper part (third) of the stomach were seen more frequently only in Group B patients with a percentage ratio of 7.5%. The tumor arising from the middle third of the stomach was found to be 10.8% in Group A and 11% in Group B, respectively. The tumor arising from the whole stomach was found only in Group B patients with a percentage ratio of 4.2%. With regard to histologic classification, the predominance of undifferentiated tumors found in both groups was remarkable. There were 44 patients (95.6%) in Group A and 110 patients (92.4%) in Group B, although these differences were statistically not significant (*P* = 0.573). Concerning the depth of tumor invasion, most patients in both groups were diagnosed in T4 stage (*P* = 0.027). There were a total of 23 patients in Group A with nodal involvement; Stage IIIC was most frequently found in both groups (*P* = 0.027), but statistically, there were no significant differences observed between Group A and Group B patients regarding TNM staging. The percentage ratio of distal gastrectomy performed was 100% with respect to Group A and 77.3% with respect to Group B. Total gastrectomy and proximal gastrectomy solely performed in Group B patients were 16% and 6.7%, respectively.

### 3.2. Follow-Up Results

**Figure 1:**
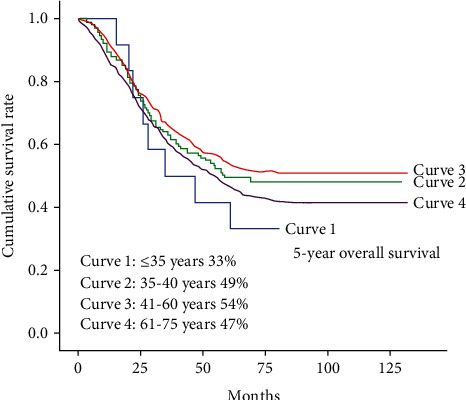
Survival curves among all the patients in four subgroups. The Kaplan–Meier Gastric Cancer survival curve based on significant prognostic predictors for overall survival in the study group patients. Survival curves among all the patients of four subgroups, which are age ≤35 years (Curve 1), age 35-40 years (Curve 2), age 41-60 years (Curve 3), and age 61-75 years (Curve 4). There was a significant difference in survival between Curve 3 and Curve 4 (*P* = 0.007). There was no significant difference in survival between other curves. The 5-year overall survival rates with respect to the young, middle, and old age group were 46%, 48%, and 39%, respectively, but when we tally the subgroups of the young age with each other, the result shows a marked variation in the overall 5-year survival rate with respect to that of subgroup A and subgroup B with 33% and 49% survival outcomes.

At the time of analysis, the total follow-up rate is 1156/1379 (83.8%), and the median follow-up duration was 53 months (range: 1-132 months). The follow-up rate was expressed as 145/165 (87.9%) for YG, 595/716 (83.1%) for MG, and 416/498 (83.5%) for OG (*P* = 0.315). The 5-year overall survival rate with respect to the young, middle, and old age groups were 46%, 48%, and 39%, respectively (Figure 1). The univariate and multivariate analyses of various clinicopathological factors associated with surgical outcomes are presented in Table 5. In the univariate analysis, tumor location, Bormann type, pathological grade, tumor size, tumor stage, nodal involvement, and positive resection degree significantly affected the prognosis. No significant difference in the overall survival duration was observed according to sex. The multivariate analysis revealed that independent negative prognostic factors were middle tumor location (L), *P* = 0.016, OR = 0.795, 95%CI = [0.659; 0.959]; larger tumor size, *P* = 0.026, OR = 1.727, 95%CI = [1.067; 2.797]; positive resection margin, *P* < 0.001, OR = 2.167, 95%CI = [1.751; 2.682]; tumor stage (T4), *P* < 0.001, OR = 2.572, 95%CI = [1.709; 3.870]; and nodal involvement N1, *P* = 0.005, OR = 1.506, 95%CI = [1.123; 2.020]; N2, *P* < 0.001, OR = 1.708, 95%CI = [1.289; 2.263]; and N3, *P* < 0.001, OR = 2.986, 95%CI = [2.314; 3.854].

## 4. Discussion

Gastric cancer is usually a disease of the aged, with a mean age of approximately 50 to 60 years [1–4]. Up to 10% of patients with gastric cancer have a family history, especially in those young patients, and E-cadherin mutation. Previous researches directed towards younger patients were very less, and these studies either included a small number of patients with a contradictory result or they divided the patients into 2 groups, i.e., young vs. the rest, and compared the prognosis between the 2 groups. In contrast, we divided 1379 gastric cancer patients into 3 groups; YG, MG, and OG. YG was further divided into two groups, Group A and Group B. Nearly 5-15% of the patients with gastric carcinoma are <40 years and only 1-2% of the patients are <30 years [5, 7, 8]. As reported by Kath et al., the female patient ratio was 75% in gastric cancer patients below the age of 30 years [8]. Our result shows that 11.96% of patients were under the age of 40 years, 50.3% were female (M/F ratio 1 : 1). When the analysis was further repeated solely in the young group with utmost scrutiny, we found that 1.16% patients were under the age of 30 years, of which 62.5% were female (M/F ratio 1 : 1.6). As previously reported, our results also demonstrated that the male-to-female ratio showed a similar female predominance pattern in the YG [17–19]. We found that the occurrences of tumor lesions in the lower third of the stomach were significantly higher in YG than in the MG or OG. The YG was more likely to have histologically undifferentiated tumor compared with the MG and OG. Distal gastrectomy was more frequently performed in all age groups. The mean tumor size was smaller in YG with respect to the MG and the OG patients. The mean postoperative stay was less in YG with respect to MG and OG. According to previous studies, young patients are always diagnosed in advanced level of TNM stages [11–13, 20]. When we examined a total of 1379 cases, what we found were higher ratios of T4 invasion, N3 involvement, and stage IIIC, which is in contrast to stage IV as found by previous studies. In relation to histological types, undifferentiated and diffuse types of carcinomas were observed more frequently in the YG [17–19]. However, in our present study, tumors that were histologically undifferentiated could be seen in young patients. Our analysis suggests that a high proportion of T4 and N3 is an independent negative prognostic factor influencing cancer-related survival. Diagnostic delay could now be taken as the only probable cause in leading to a more advanced stage of tumors in young patients [21]. Therefore, an early diagnosis is very important in successfully completing a curative resection that gives a better prognosis [22]. Moreira et al. reported that tumor location, histological classification, depth of tumor invasion, nodal involvement, lymphatic invasion, vascular invasion, distant metastases, and TNM stages in univariate analysis have significant impacts on the prognosis [23]. Moreover, in the present study, tumor location, tumor size, deeper tumor invasion, advanced nodal involvement, and R1/R2 resection margin have significant negative impacts on prognosis using multivariate analysis. The clinical result of young patients with gastric cancer remains disputable. Some reports showed a similar survival rate in both young and elderly patients [10–12], whereas some studies have concluded with a better prognosis in young patients [18]. Overall, there are few reports in this unique group of patients, most of these studies are case series with a limited number of patients, and most of these reports included patients less than 40 years old [20, 24, 25]. Our study included young patients who were younger than 40 years old, and this may provide additional information to the management. We analyzed that the prognosis of gastric cancer patients was poor in young patients especially age <30 years compared to the MG and the OG.

Significant differences were observed in the prognosis between subgroup A and subgroup B patients, where the correlation between age and prognosis was determined. The main results of our investigation of the 5-year overall survival rates with respect to the young, middle, and old age group were 46%, 48%, and 39%, respectively, but when we tally the subgroups of the young age with each other, the result shows a marked variation in the overall 5-year survival rate with respect to that of subgroup A and subgroup B with 33% and 49% survival outcomes. It also shows a dramatic variation when we compare the data of the subgroups of the young age with the middle and old age groups. Even though gastric cancer was said to occur in elderly and middle-aged group, the risk factor was more in the younger generation (especially female) with a poor prognostic factor and late detection. Therefore, close scrutiny and monitoring should be done in young adults with respect to the associated common symptoms which could indicate the presence of gastric cancer at an early stage. Also, the younger generation should be educated on the signs and symptoms of the disease leading to an early detection and preventing the advancement of the disease thereby decreasing the mortality rate in the young generation. Early detection of the disease in the young generation could let them have a better prognosis. Thus, the key to improve the surgical outcome in young patients is dependent on early diagnosis and curative resection. Prevention of the disease is inevitable as gastric carcinoma is truly difficult to control once its clinical entity has been established.

### 4.1. Limitations of our Study

Our research was entirely based on a retrospective design and the exclusion of nonsurgical patients which is the major limitations of this study. Another important limitation is that the research was conducted in a tertiary hospital thereby missing the opportunity to diagnose the disease at an early stage due to the potential lack in accessibility which may have caused the major delay in treating patients and thus altering the prognosis. As our study is based on clinicopathologic characteristic, we did not focus to collect the data of nonadjuvant chemotherapy and its regimes even though most of our patients received neoadjuvant chemotherapy. Our study also lacks disease free survival, disease specific survival of patient's family history, and E-cadherin mutation information. The small number of patients in Group A making any strict statistical comparisons open to errors is related to the limited power from the small number in the YG. Nevertheless, our analysis revealed more important clinicopathological characteristics and prognosis that analyzed gastric cancer in young patients compared with middle aged and elderly. Further studies are needed to obtain the unique features and prognosis of younger patients.

## 5. Conclusion

The young age groups of patients were predominantly female and had a higher proportion of poorly differentiated and undifferentiated type of tumor; moreover, patients aged <30 years had a poor prognosis. In addition, gastric cancer can occur in patients less than 30 years old, and symptoms suggestive of gastric cancer should be investigated aggressively; therefore, a close screening for E-cadherin scrutiny and family history monitoring should be done in younger patients especially those associated with high-risk factors which could indicate the presence of the disease at an early stage.

## Figures and Tables

**Table 1:** Clinicopathological details and surgical results of gastric carcinoma in young, middle, and old age group patients.

Characteristics		Young group *N* = 165 (11.96%)	Middle group *N* = 716 (51.92%)	Old group *N* = 498 (36.11%)	*P* value
Age	Years	35.4 ± 3.9	52.0 ± 5.9	66.6 ± 3.9	<0.001

Sex	Male	82 (49.7)	511 (71.4)	382 (76.7)	<0.001
	Female	83 (50.3)	205 (28.6)	116 (23.3)	

Resection margin	R0	139 (84.2)	640 (89.4)	427 (85.7)	0.070
	R1 and R2	26 (15.8)	76 (10.6)	71 (14.3)	

Resection patterns	DG	123 (74.5)	429 (59.9)	260 (52.2)	<0.001
	TG	29 (17.6)	164 (22.9)	128 (25.7)	
	PG	13 (7.9)	128 (25.7)	110 (22.1)	

Tumor size	cm	4.8 ± 2.7	5.1 ± 2.8	5.4 ± 3.0	0.042

Tumor location	U	19 (11.5)	171 (23.9)	152 (30.5)	<0.001
	M	24 (14.5)	93 (13.0)	68 (13.7)	
	L	117 (70.9)	439 (61.3)	268 (53.8)	
	UML	5 (3.0)	13 (1.8)	10 (2.0)	

Macroscopic type	EGC	33 (20.0)	124 (17.3)	69 (13.9)	0.346
	Types 1-2	78 (47.3)	330 (46.1)	235 (47.2)	
	Types 3-4	54 (32.7)	262 (36.6)	194 (39.0)	

Differentiate degree	G1 and G2	15 (9.1)	100 (14.0)	100 (20.0)	0.001
	G3 and G4	150 (90.9)	616 (86.0)	398 (79.9)	

Postoperative stay	Days	9.8 ± 2.5	10.5 ± 4.5	11.5 ± 5.6	<0.001

DG: distal gastrectomy; TG: total gastrectomy; PG: proximal gastrectomy; U: upper; M: middle; L: lower; EGC: early gastric cancer.

**Table 2:** TNM stages of the young, middle, and old age group patients.

	Characteristics	Young group *N* = 165 (11.96%)	Middle group *N* = 716 (51.92%)	Old group *N* = 498 (36.11%)	*P* value
T stage	T1	33 (20.0)	124 (17.3)	69 (13.9)	0.049
	T2	16 (9.7)	81 (11.3)	52 (10.4)	
	T3	6 (3.6)	12 (1.7)	13 (2.6)	
	T4	110 (66.7)	499 (69.7)	364 (73.1)	

N stage	N0	48 (29.1)	228 (31.8)	160 (32.1)	0.453
	N1	24 (14.5)	124 (17.3)	95 (19.1)	
	N2	33 (20.0)	121 (16.9)	96 (19.3)	
	N3	60 (36.4)	243 (33.9)	147 (29.5)	

TNM stage	IA	25 (15.2)	93 (13.0)	61 (12.2)	0.327
	IB	9 (5.5)	58 (8.1)	28 (5.6)	
	IIA	11 (6.7)	24 (3.4)	24 (4.8)	
	IIB	17 (10.3)	103 (14.4)	75 (15.1)	
	IIIA	14 (8.5)	88 (12.3)	69 (13.9)	
	IIIB	27 (16.4)	103 (14.4)	69 (13.9)	
	IIIC	40 (24.2)	172 (24.0)	109 (21.9)	
	IV	22 (13.3)	75 (10.5)	63 (12.7)	

Positive lymph nodes	Number	6.6 ± 8.5	6.3 ± 8.4	5.3 ± 7.1	0.215

Harvested lymph nodes	Number	23.6 ± 12.7	24.3 ± 13.9	22.2 ± 13.6	0.028

DG: distal gastrectomy; TG: total gastrectomy; PG: proximal gastrectomy; U: upper; M: middle; L: lower; EGC: early gastric cancer.

**Table 3:** Clinicopathological characteristics and surgical results of the young patients with two subgroups.

	Characteristics	Young group = 165		*P* value
		Age ≤35 years *N* = 46	Age 35-40 years *N* = 119	
Sex	Male	21	61	0.048
	Female	35	58	

Resection margin	R0	43	99	0.496
	R1 and R2	3	20	

Resection patterns	DG	46	92	0.047
	TG	0	19	
	PG	0	8	

Tumor size	cm	4.8 ± 2.1	4.8 ± 2.8	0.05

Tumor location	U	0	9	0.459
	M	5	13	
	L	41	92	
	UML	0	5	

Macroscopic type	EGC	14	19	0.123
	Types 1-2	16	62	
	Types 3-4	16	38	

Differentiate degree	G1 and G2	2	9	0.573
	G3 and G4	44	110	

Postoperative stay	Days	9.5 ± 1.5	9.8 ± 2.5	0.927

DG: distal gastrectomy; TG: total gastrectomy; PG: proximal gastrectomy; U: upper; M: middle; L: lower; EGC: early gastric cancer.

**Table 4:** TNM stages of the young patients with two subgroups.

	Characteristics	Young group *N* = 165		*P* value
		Age ≤35 years *N* = 46	Age 35-40 years *N* = 119	
T stage	T1	13	18	0.027
	T2	5	8	
	T3	5	2	
	T4	23	91	

N stage	N0	10	34	0.061
	N1	3	18	
	N2	3	27	
	N3	30	40	

TNM stage	IA	9	17	0.027
	IB	2	6	
	IIA	2	10	
	IIB	2	11	
	IIIA	2	10	
	IIIB	2	21	
	IIIC	23	29	
	IV	4	15	

Positive lymph nodes	Number	8.4 ± 7.1	6.4 ± 8.6	0.443

DG: distal gastrectomy; TG: total gastrectomy; PG: proximal gastrectomy; U: upper; M: middle; L: lower; EGC: early gastric cancer.

**Table 5:** Univariate and multivariate predictors of overall survival.

Variables	Univariate OR (95% CI)	*P* value	Multivariate OR (95% CI)	*P* value
Age				
≤35	1			
35-40	0.728 [0.349; 1.515]	0.395		
41-60	0.663 [0.328; 1.339]	0.663		
61-75	0.839 [0.415; 1.698]	0.839		

Sex				
Male	1			
Female	0.993 [0.831; 1.188]	0.942		

Resection margin				
R0	1		1	
R1/R2	3.860 [3.167; 4.703]	<0.001	2.167 [1.751; 2.682]	<0.001

Tumor location				
U	1		1	
M	0.817 [0.624; 1.070]	0.142	0.778 [0.588; 1.012]	0.061
L	0.692 [0.575; 0.832]	<0.001	0.795 [0.659; 0.959]	0.016
UML	3.913 [2.480; 6.173]	<0.001	1.389 [0.844; 2.286]	0.196

Macroscopic type				
EGC	1			
Types 1-2	4.129 [2.848; 5.987]	<0.001		
Types 3-4	6.337 [4.371; 9.186]	<0.001		

Differentiate degree				
G1 and G2	1			
G3 and G4	1.654 [1.278; 2.140]	<0.001		

Tumor size (cm)				
0-2	1		1	
2-5	2.456 [1.664; 3.626]	<0.001	1.307 [0.869; 1.965]	0.198
5-8	4.485 [3.026; 6.648]	<0.001	1.615 [1.058; 2.463]	0.026
>8	7.852 [5.090; 12.112]	<0.001	1.727 [1.067; 2.797]	0.026

T stage				
T1	1		1	
T2	1.638 [1.003; 2.675]	<0.048	1.125 [0.680; 1.863]	0.647
T3	3.230 [1.659; 6.289]	<0.001	2.091 [1.059; 4.127]	0.034
T4	5.884 [4.093; 8.459]	<0.001	2.572 [1.709; 3.870]	<0.001

N stage				
N0	1		1	
N1	2.002 [1.505; 2.664]	<0.001	1.506 [1.123; 2.020]	0.005
N2	2.651 [2.032; 3.460]	<0.001	1.708 [1.289; 2.263]	<0.001
N3	5.552 [4.405; 6.997]	<0.001	2.986 [2.314; 3.854]	<0.001

DG: distal gastrectomy; TG: total gastrectomy; PG: proximal gastrectomy; U: upper; M: middle; L: lower; EGC: early gastric cancer, OR: odds ratio; 95% CI: confidence interval.

## Data Availability

The data used to support the findings of this study are available from the corresponding author upon request.

## Abbreviations

YG: Young age group
MG: Middle age group
OG: Elderly group.
